# Ultrasound-triggered dual-drug release from poly(lactic-co-glycolic acid)/mesoporous silica nanoparticles electrospun composite fibers

**DOI:** 10.1093/rb/rbv019

**Published:** 2015-10-14

**Authors:** Botao Song, Chengtie Wu, Jiang Chang

**Affiliations:** State Key Laboratory of High Performance Ceramics and Superfine Microstructure, Shanghai Institute of Ceramics, Chinese Academy of Sciences, 1295 Dingxi Road, Shanghai 200050, People’s Republic of China

**Keywords:** electrospinning, mesoporous silica, dual drug release, ultrasound irradiation

## Abstract

The aim of this study was to achieve on-demand controlled drug release from the dual-drug-loaded poly(lactic-co-glycolic acid)/mesoporous silica nanoparticles electrospun composite fibers by the application of ultrasound irradiation. Two drugs were loaded in different part of the composite fibrous materials, and it was found that ultrasound as an external stimulus was able to control release of drugs due to both its thermal effect and non-thermal effect. With the selective irradiation of ultrasound, the drug carrier enabled to realize controlled release, and because of different location in fibers and sensitivity of two different kinds of drugs to ultrasound irradiation, the release rate of two drugs was different. These results indicated that ultrasound irradiation was a facile method to realize the on-demand controlled release of two drugs from the electrospun fibers.

## Introduction

Recently, study on dual-drug delivery systems has been reported to obtain improved therapeutic effects [1–3]. In comparison with the single-drug delivery system, the dual-drug delivery system, which is capable of releasing two drugs with different therapeutic functions, may not only improve therapeutic efficiency but also minimize negative side effects of drugs [4]. However, the control of the release of two different drugs on-demand of dosage, duration and timing is still a big challenge [5], which is often required clinically [6, 7]. For example, diabetic patients always need insulin to manage blood glucose [8]. However, after several times of treatment, the insulin is in short supply. This problem can be solved by combining the therapeutic effects of insulin and cyclic adenosine monophosphate (cAMP), because cAMP can stimulate pancreas beta cells to secrete insulin continuously [9–11]. Therefore, before eating, the diabetic patients only need a small amount of insulin and cAMP to control blood glucose concentration, while more insulin and cAMP are required after meals due to the significant increase of the blood glucose concentration.

**Figure 1.**
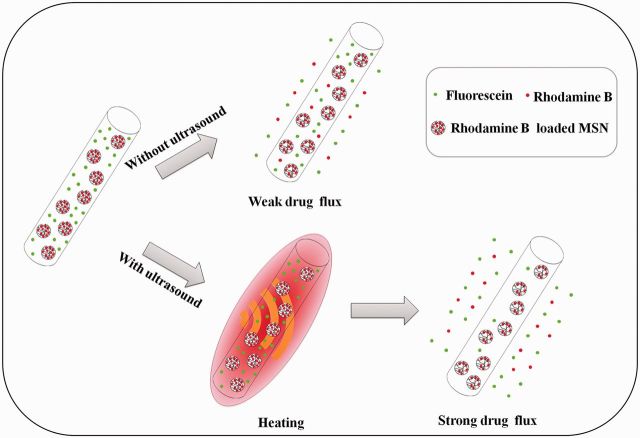
A schematic for the drug release profiles of RHB and FLU from dual-drug-loaded PLGA/FLU/RHB-loaded MSNs electrospun fiber when treating with and without ultrasound

Recent studies have demonstrated that the electrospun fiber is a promising localized drug delivery carrier [12–14], and the dual-drug-loaded electrospun fibers have been fabricated by some groups in different ways [15, 16]. Su *et al*. [15] used the coaxial electrospinning to prepare poly(L-lactide-co-caprolactone)/collagen nanofibers and load materials with bone morphogenetic protein and dexamethasone for bone tissue engineering applications. Recently, we have reported fabrication of dual-drug-loaded poly(lactic-co-glycolic acid)/mesoporous silica nanoparticles (PLGA/MSNs) electrospun composite fibers with one drug directly embedded into the PLGA matrix for fast release and the other in the MSNs for sustained release [17]. Although the above-mentioned dual-drug-loaded electrospun fibers can release two drugs in different rates at different timing, the control of the release of drugs is still not optimal and the release of individual drug could not be controlled on-demand. However, in our preliminary study, we have noticed that the release of drugs from PLGA/MSNs electrospun composite fibers is affected by temperature. On the basis of this observation, we propose the hypothesis that the individual release of two different drugs may be controlled by control of the temperature, and ultrasonic stimulation might be used to realize the temperature controlled drug release. Ultrasonically triggered drug delivery system is of special interest in biomedical fields because it is non-invasive or minimally invasive, and the ultrasound can not only trigger drug release from the drug carriers but also enhance the permeation of drugs into the deep body with minimum thermal damage to the surrounding tissue [18]. Therefore, the goal of this study is to investigate the effect of heating on the release of the two drugs from PLGA/MSNs composite fibers, more importantly, to study the possibility to control the on-demand release of two drugs by ultrasound irradiation (Fig. 1).

## Materials and methods

### Materials

N,N-Dimethylformamide, tetrahydrofuran, rhodamine B (RHB) and fluorescein (FLU) were all purchased from Sinopharm Chem. Reagents Co., Ltd. PLGA (*M*_W_ = 150 000, lactic acid:glycolic acid = 90:10) was purchased from Jinan Daigang Bio-technology Co., Ltd. China. All chemicals were used as received without further purification.

### 2.2 Fabrication of drug-loaded electrospun mat

The procedures for synthesis of MSNs and RHB-loaded MSNs were the same as we previously reported, and the loading capacity of MSNs for RHB was 24.26 mg/g [19]. The PLGA/FLU/RHB-loaded MSNs electrospun composite mat loading with 5% of FLU (with respect to the weight of PLGA) and 25% of RHB-loaded MSNs (with respect to the weight of PLGA) was fabricated. First, 0.1375 g FLU was added into the mixed solvent of 7.5 ml of N,N-dimethylformamide and 2.5 ml of tetrahydrofuran, followed by adding 0.6815 g RHB-loaded MSNs. After ultrasound dispersion for 30 s, 2.6739 g PLGA was then added into the above suspension with vigorous stirring until PLGA was fully dissolved. Then, the suspension was transferred into a syringe for further electrospinning. A 13.0 kV positive voltage was applied to the solution via a needle, and the flow rate was fixed at 3.0 ml/h. A drum with the rotating speed of 450 rpm was used for fiber collection, and the distance between the needle and the collector was 12.0 cm. PLGA/FLU electrospun mat and PLGA/RHB-loaded MSNs electrospun mat were both prepared by the similar method.

### Characterization

The electrospun fibers were observed by using scanning electron microscopy (SEM, S-4800, HITACHI, Japan). The average diameter and diameter distribution of the fibers were analyzed by image analysis software (Nano Measurer 1.2). Fluorescent images were recorded by using a laser scanning confocal microscopy (FluoViewTM FV1000, Olympus, Japan).

### Temperature-triggered drug release

The centrifuge tube loaded with 50 ml phosphate-buffered saline (PBS, pH = 7.4) was placed into preheated water bath at 30°C, 37°C and 43°C, respectively. To have a better comparison between the RHB release profiles from PLGA/FLU/RHB-loaded MSNs electrospun mat, PLGA/RHB-loaded MSNs electrospun mat and the control RHB-loaded MSNs, the total amount of the drug RHB in three kinds of samples was set as the same. Therefore, 20 mg of the PLGA/FLU/RHB-loaded MSNs electrospun mat and 19.23 mg of the PLGA/RHB-loaded MSNs electrospun mat and 3.85 mg of the RHB-loaded MSNs containing the same weight of RHB (0.093 mg) were used for the drug release experiments. Similarly, to assess the FLU release profiles, 20 mg of the PLGA/FLU/RHB-loaded MSNs electrospun mat and 16.15 mg of the PLGA/FLU electrospun mat with the same FLU amount were used. At selected time intervals, 1 ml of the buffer solution was removed for analysis and replaced with 1 ml of fresh buffer solution. The absorbance of FLU and RHB in the buffer solution was measured by a microplate spectrophometer at the wavelength of 491 nm and 553 nm, respectively. The measured absorbance was converted to concentration based on the standard curves. All drug release experiments were repeated at least three times.

### Ultrasound-triggered drug release

For the ultrasound-triggered drug release, a 25 kHz low-frequency ultrasonic sonochemistry-assisted microwave reactor (XO-SM50, Nanjing Xianou Instrument Manufacturing Co., Ltd., China) was used. PBS (50 ml) was preheated to 30°C by the ultrasound with a power of 10 W and a pulsed cycle of 2 s on and 2 s off, and then the drug carriers were quickly put into the buffer solution. The buffer solution was replaced with fresh solution at predetermined time intervals, and the concentration of the two model drugs was determined using a microplate spectrophometer mentioned above. In addition, the temperature of the buffer solution at different time intervals was also monitored and recorded by a temperature probe inserted in the buffer solution.

For the control experiments, to avoid the thermal effect caused by the ultrasound irradiation promoting the drug release, a circulating water bath was used throughout the experiments to maintain the temperature of the buffer solution at 30°C.

The ultrasound with different powers (10 W, 20 W and 30 W) and different pulsed cycles (2 s on and 1 s off, 2 s on and 2 s off and 2 s on and 4 s off) were used to investigate the effect of ultrasonic powers and pulsed cycles on the drug release behaviors, respectively.

Finally, to realize the on-demand release, the dual-drug-loaded PLGA/FLU/RHB-loaded MSNs electrospun mat was firstly incubated in the water bath (30°C) for 30 min and then treated with ultrasound for another 30 min.

### Statistical analysis

All data were expressed as means ± standard deviation and were analyzed by a Student’s *t*-test, and a *P*-value < 0.05 was considered statistically significant.

## Results and discussion

### The morphology of electrospun fiber

**Figure 2.**
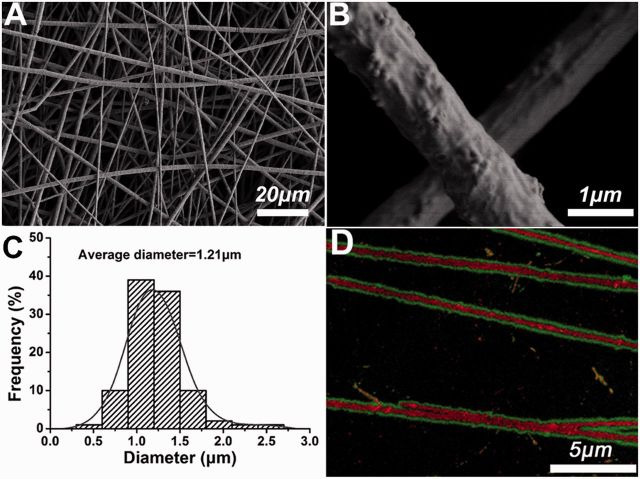
(**A**, **B**) SEM images, (**C**) fiber diameter distribution and (**D**) the laser scanning confocal microscopy images of dual-drug-loaded PLGA/FLU/RHB-loaded MSNs electrospun fibers

Figure 2 showed SEM images and diameter distribution of PLGA/FLU/RHB-loaded MSNs electrospun fibers. It was obviously observed that the fibers had uniform structure. Similar to our previous results [17], some RHB-loaded MSNs were located on the fiber surface forming the protrusions. In addition, the average diameter of the fibers was measured, which was 1.21 μm. The laser scanning confocal microscopy was used to visualize the distribution of the two model drugs FLU and RHB in the electrospun fibers. As seen from Fig. 2D, PLGA/FLU/RHB-loaded MSNs electrospun fibers exhibited two different colors. RHB-loaded MSNs was located in the center part of the fiber (the red color), and FLU was distributed in the fiber surrounding red-colored MSNs (the green color), which demonstrated that no detective RHB was found to diffuse from MSNs when preparation of the spinning solution, and RHB was well encapsulated within the pores of MSNs.

### Effect of temperature on the drug release behaviors

**Figure 3.**
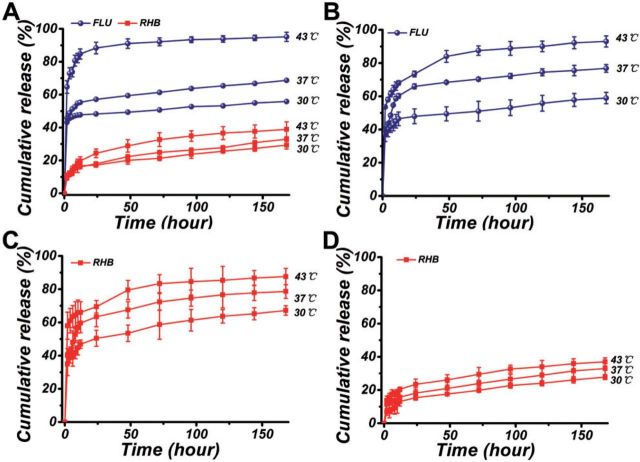
The cumulative release of FLU and RHB from (**A**) PLGA/FLU/RHB-loaded MSNs electrospun mat, (**B**) PLGA/FLU electrospun mat, (**C**) RHB-loaded MSNs and (**D**) PLGA/RHB-loaded MSNs electrospun mat at different temperatures (30, 37, 43°C)

Figure 3A showed the drug release profiles of FLU and RHB from PLGA/FLU/RHB-loaded MSNs electrospun mat at different temperatures (30, 37 and 43°C). As observed from the results, FLU showed a rapid release manner and RHB released in a sustainable way. After releasing for 168 h, the cumulative release of FLU was about 55.81 ± 0.28, 68.74 ± 0.53 and 95.16 ± 2.79% at the temperature of 30, 37 and 43°C, respectively. And the RHB released about 29.32 ± 2.32, 32.96 ± 1.00 and 38.93 ± 4.55%, respectively. These results indicated that the release of both FLU and RHB could be promoted by increasing the temperature of the buffer solution, and the temperature had a relative larger impact on the release of FLU than RHB.

The release behaviors of PLGA/FLU electrospun mat, RHB-loaded MSNs and PLGA/RHB-loaded MSNs electrospun mat at different temperatures were also investigated. It was found that FLU in PLGA/FLU electrospun mat showed similar release behaviors as it in PLGA/FLU/RHB-loaded MSNs electrospun mat. It was totally released about of 58.95 ± 3.41, 76.83 ± 2.31 and 93.02 ± 3.29%, respectively (Fig. 3B). For RHB, with the increase of temperature from 30 to 43°C, the release of RHB from RHB-loaded MSNs could reach to 67.21 ± 2.85, 78.65 ± 4.12 and 87.59 ± 4.92%, respectively (Fig. 3C); and RHB release from PLGA/RHB-loaded MSNs electrospun mat was about 27.84 ± 1.51, 32.93 ± 3.65 and 36.98 ± 2.41% (Fig. 3D), which was similar with the release from PLGA/FLU/RHB-loaded MSNs electrospun mat.

The results suggested that the release profiles of two drugs could be controlled by temperature, and the temperature had a greater influence on the release of FLU than RHB. It was known that polymers were unusually thermally unstable, and it was no exception with the polymer PLGA used in our study. When the temperature of the buffer solution was maintained at 30°C, the polymer chains were frozen [20], so the gap between the polymer chains was small, which was not in favor of the release of FLU. After the temperature increased to 43°C, which was near the glass transition temperature of PLGA [21–23], and the flexibility of the chains increased substantially and the gap between the molecular chains became larger, which promoted the release of FLU. Moreover, in this study, the polymer PLGA used in this study had a very high molecule weight about 150 000 Dalton and contains 90% of the monomer LA, which decided the stable structure of the used polymer PLGA with slow degradation. In addition, the drug release experiment was carried out in a short period of 168 h, such a short time period could not distinctively lead to the PLGA degradation. In our previous study, we had carefully investigated the morphology change and the mass loss of the drug-loaded electrospun mats before and after soaking in PBS [17]. It was found that the morphology of the electrospun fibers was not significantly changed and there was no apparent mass loss of electrospun mats after soaking in PBS for 324 h. Therefore, it was speculated that thermal motion of PLGA played a key role to influence drug delivery, and the degradation rate of PLGA is not the major factor for drug release. When compared with PLGA, MSNs had very good thermal stability [24]. As the temperature of buffer solution increased, the structure of MSNs would not be changed and only thermal motion of the RHB molecules became quicker, which would slightly speed up the diffusion of RHB from the MSNs into the solution environment.

### Effect of ultrasound irradiation on the drug release behaviors

As mentioned above, the release profiles of two drugs could be controlled by temperature. It is known that ultrasound has heat generating effect. Therefore, it is reasonable to assume that ultrasound irradiation may be used to further control the dual-drug release from PLGA/FLU/RHB-loaded MSNs electrospun composite mat.

**Figure 4.**
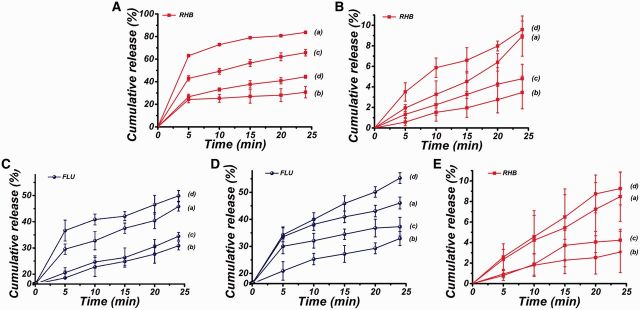
The release profiles of RHB and FLU from (**A**) RHB-loaded MSNs, (**B**) PLGA/RHB-loaded MSNs electrospun mat, (**C**) PLGA/FLU electrospun mat and (**D**, **E**) PLGA/FLU/RHB-loaded MSNs electrospun mat under different release conditions (a: Ultrasound irradiation; b: Constant temperature 30°C; c: Ultrasound irradiation at constant temperature 30°C; d: Constant temperature 43°C)

Figure 4A showed the release behaviors of RHB-loaded MSNs in different release environments. The release of RHB with and without ultrasound irradiation was firstly measured, and after exposed to ultrasound radiation, the final temperature of buffer solution increased to 43°C. As seen from Fig. 4A(a), about 84 ± 1.03% of RHB was released after irradiation by ultrasound, which was much higher than that without ultrasound irradiation (about 31 ± 5.05%, Fig. 4A(b)). The results indicated that ultrasonic irradiation could enhance the release of RHB from RHB-loaded MSNs.

It had previously been reported that thermal and non-thermal effects were the two main mechanisms for ultrasound-enhanced drug release [25, 26]. In our study, it was also observed that the temperature of the buffer solution gradually increased after irradiation by ultrasound irradiation with a power of 10 W and a pulsed cycle of 2 s on and 2 s off (Fig. 8A). To find out the dominating factor for the ultrasound-enhanced drug release, the temperature of the buffer solution was kept at 30°C throughout the experiment to prevent heat-induced drug release, then the ultrasound was introduced to investigate the effect of non-thermal effect on drug release. The result showed that the cumulative release of RHB with ultrasonic treatment at 30°C was 65.70 ± 2.89% (Fig. 4A(c)). From the equations below, both thermal-induced drug release and non-thermal induced drug release could be calculated to determine the dominating effect for the enhanced release.

$$\begin{array}{l} Y=X-X_{\text{1}}\\ Y_{\text{1}}=X_{\text{2}}-X_{\text{1}}\\ Y_{\text{2}}=Y-Y_{\text{1}} \end{array}$$

Where *Y*, *Y*_1_ and *Y*_2_ represented the increased percentage of drug release, the increased percentage of drug release caused by non-thermal effect and the increased percentage of drug release caused by thermal effect, respectively. *X,* *X*_1_ and *X*_2_ represented the cumulative release with ultrasound irradiation, the cumulative release at constant temperature (30°C) without ultrasound irradiation and the cumulative release with ultrasound irradiation at 30°C, respectively.

The data were summarized and listed in Table 1. The results indicated that the non-thermal effect was the main mechanism for the enhanced drug release from RHB-loaded MSNs. The ultrasound energy was transmitted through the buffer solution to RHB-loaded MSNs, making RHB-loaded MSNs vibrated acutely, thus, RHB could be easily released from MSNs [27].

**Table 1.** The total released percentage of FLU and RHB from RHB-loaded MSNs, PLGA/RHB-loaded MSNs electrospun mat, PLGA/FLU electrospun mat and PLGA/FLU/RHB-loaded MSNs electrospun mat in the different release conditions and the increased percentage caused by non-thermal effect and thermal effect

	RHB released from RHB-loaded MSNs (%)	RHB released from PLGA/RHB-loaded MSNs mat (%)	FLU released from PLGA/FLU mat (%)	FLU released from PLGA/FLU/RHB-loaded MSNs mat (%)	RHB released from PLGA/FLU/RHB-loaded MSNs mat (%)
30°C	30.72	3.46	30.82	32.91	3.08
Ultrasound irradiation	83.72	8.95	45.81	46.05	8.48
Ultrasound irradiation at 30°C	65.70	4.80	34.47	37.30	4.23
The increased percentage	53.0	5.49	14.99	13.14	5.40
Contributed by non-thermal effect	34.98	1.34	3.65	4.39	1.15
Contributed by thermal effect	18.02	4.15	11.34	8.75	4.25

The release of RHB-loaded MSNs at constant temperature 43°C without ultrasound treatment was further evaluated, which was slower than that with ultrasound treatment as seen from Fig. 4A(d). This further proved that non-thermal effect was main mechanism for the enhanced drug release.

RHB-loaded MSNs were then incorporated into the polymer matrix to form the PLGA/RHB-loaded MSNs electrospun mat, and the release behaviors of the electrospun composite mat in the different release environments were further investigated (Fig. 4B). In the absence of ultrasound irradiation, the cumulative release of RHB from the composite mat was 3.46 ± 1.59%. In contrast, after treatment with ultrasound, RHB released faster and the cumulative release could reach to 8.95 ± 1.96%. Based on these results, it was indicated that, when RHB was carried in MSNs, which were located in PLGA polymer fibers, ultrasound could also enhance the RHB release from PLGA/RHB-loaded MSNs electrospun mat. The cumulative release of RHB with ultrasonic treatment at constant temperature 30°C was then conducted to find out the mechanism for the enhanced drug release. According to the equations mentioned above, it was found that the thermal effect played a more important role than non-thermal effect in enhancing drug release from PLGA/RHB-loaded MSNs electrospun mat (Table 1). The release profile of PLGA/RHB-loaded MSNs electrospun mat at the constant temperature of 43°C without ultrasound irradiation was further evaluated, and it was found that the cumulative release was larger than that with ultrasound treatment, which also confirmed that thermal effect was more important for the enhanced drug release in the composite materials. Although in pure RHB-loaded MSNs, the non-thermal effect was the key factor; after incorporation into the PLGA fibers, RHB-loaded MSNs was tightly confined in the polymer matrix and they could not be effectively stimulated when exposed to ultrasound. Thus, the heating effect became the dominating effect in drug release. The FLU release kinetics from PLGA/FLU electrospun mat in different release environments were also measured (Fig. 4C), and it was indicated that the thermal effect was the dominating factor in enhancing drug release from PLGA/FLU electrospun mat (Table 1).

**Figure 5.**
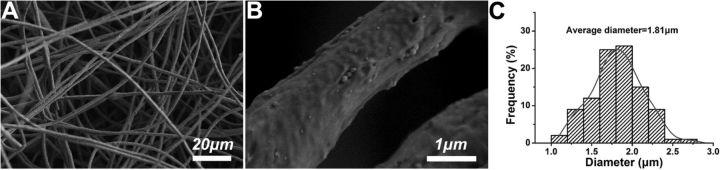
SEM images and fiber diameter distribution of dual-drug-loaded PLGA/FLU/RHB-loaded MSNs electrospun fibers after exposed to ultrasound irradiation

Finally, the release profiles of dual-drug-loaded PLGA/FLU/RHB-loaded MSNs electrospun mat in different release environments were tested. It was found that ultrasound could promote both the release of FLU and RHB (Fig. 4D and E). In addition, the data in Table 1 demonstrated that the heating effect was the key factor in promoting the release of the two drugs from the composite materials, which was different as the release of RHB from MSNs, in which ultrasound played a dominant role. The drug release experiment of the dual-drug-loaded electrospun mat at the constant temperature of 43°C was also studied, and the result indicated that the amount of cumulative release of RHB and FLU were both larger than that with ultrasound irradiation. Moreover, the morphology of the electrospun composite mat after ultrasound treatment was also observed. As seen from Fig. 5, the irradiation of ultrasound did not destroy the structure of the composite fiber, and no fractured fibers were observed and no defects were formed on the fiber. The increased average of diameter of dual-drug-loaded PLGA/FLU/RHB-loaded MSNs electrospun fibers after exposed to ultrasound irradiation was due to the relaxation of polymer chain. During the process, the water molecules diffused into the fibers and decreased the interaction between the polymer chains, which led to the chains in a relaxed state, and the fiber swelled and the average diameter of the fibers increased [28].

### Effect of ultrasonic powers and pulsed cycles on the drug release behaviors

**Figure 6.**
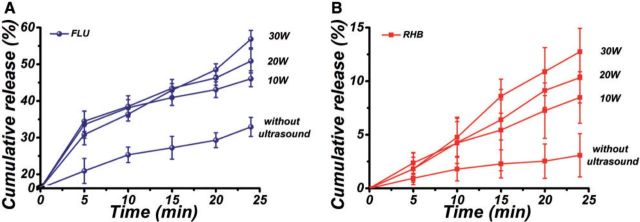
The release profiles of (**A**) FLU and (**B**) RHB from PLGA/FLU/RHB-loaded MSNs electrospun mat when treated without ultrasound and irradiated by ultrasound with different powers (10 W, 20 W, 30 W)

Figure 6 showed the release profiles of FLU and RHB from PLGA/FLU/RHB-loaded MSNs electrospun mat with different powers of ultrasound irradiation. It was observed that FLU and RHB gradually released from the drug-loaded electrospun mat in the case without ultrasound irradiation. In contrast, with ultrasound irradiation, the release of the two drugs was both greatly enhanced, and with the increase of the ultrasonic power from 10 W to 30 W, the cumulative release of FLU reached 46.05 ± 2.20, 50.88 ± 3.24 and 56.85 ± 2.39%, respectively, and the released percentage of RHB were 8.48 ± 2.40, 10.32 ± 2.35 and 12.74 ± 2.18%, respectively, which were significantly higher than that without irradiation (*P* < 0.05). These results suggested that the cumulative release of FLU and RHB increased with the increase of ultrasonic power.

**Figure 7.**
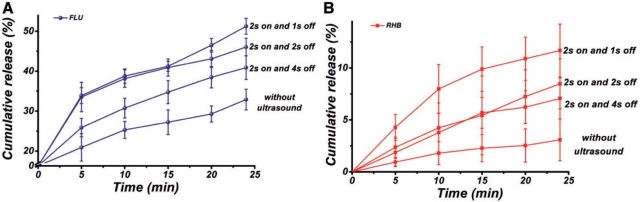
The release profiles of (**A**) FLU and (**B**) RHB from PLGA/FLU/RHB-loaded MSNs electrospun mat with different pulsed cycles of ultrasound irradiation (2 s on and 1 s off, 2 s on and 2 s off and 2 s on and 4 s off). The release of drugs without ultrasound irradiation was the control

Figure 7 showed the drug release profiles of the composite mat with different pulsed cycles of ultrasound irradiation at 10 W power. Compared with the drug release without ultrasound treatment, the release rates of the two drugs were both improved with ultrasound irradiation with different pulsed cycles. In addition, the release rate of FLU and RHB both decreased with the increase of the ‘ultrasound off’ duration from 1 s to 4 s. The above results demonstrated that the release profiles of the two drugs could be controlled by varying the power and the pulsed cycle of ultrasound irradiation.

**Figure 8.**
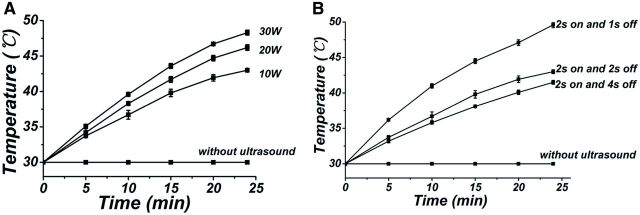
The temperature change of the buffer solution when treated without ultrasound and irradiated by ultrasound with different ultrasonic powers (**A**) and different pulsed cycles with an ultrasonic power of 10 W (**B**)

Figure 8 showed the temperature change of the buffer solution when treated with ultrasound irradiation at different powers and different pulsed cycles, respectively. It was found that the rate of temperature rise increased with the increase of ultrasound powers and decreased with the increase of ‘ultrasound off’ duration. As mentioned before, the mechanism of drug release by ultrasound irradiation was mainly attributed to the heating effect. Therefore, higher power and shorter ‘ultrasound off’ duration would cause a higher temperature and finally lead to a faster drug release. Therefore, ultrasound with different irradiation powers and different pulsed cycles could be used to modulate the temperature of the buffer solution and further to control the release of two drugs.

### The on-demand drug release

**Figure 9.**
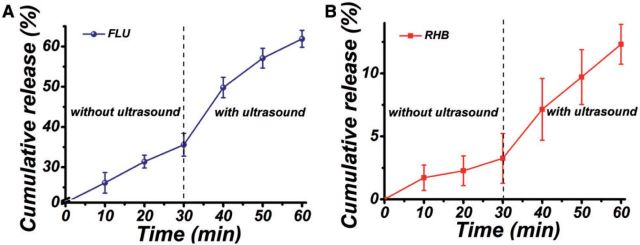
The cumulative release of (**A**) FLU and (**B**) RHB from PLGA/FLU/RHB-loaded MSNs electrospun mat first at constant temperature 30°C for 30 min and followed with the irradiation of ultrasound for another 30 min

Figure 9 showed the release profiles of FLU and RHB from dual-drug-loaded electrospun mat first without ultrasound treatment for 30 min, followed by ultrasound irradiation for another 30 min. It was found that after incubation in the silent condition (without ultrasound) for the first 30 min, the cumulative release of RHB was only 3.26 ± 1.98%. However, when exposed to ultrasound, the release rate was sharply increased and the cumulative release of RHB reached to 12.31 ± 1.58%, which was about 3.8 times high as that without ultrasound irradiation. In addition, it was observed that after ultrasound irradiation the cumulative release of FLU was also enhanced and the release percentage was about 1.74 times high as that before irradiation. The above results indicated that the on-demand drug release could be easily achieved by selective irradiation of ultrasound.

## Conclusions

In this study, we presented a controllable and on-demand drug delivery system based on the dual-drug-loaded PLGA/FLU/RHB-loaded MSNs electrospun composite fibers triggered by both ultrasound and temperature. It was found that both temperature and ultrasound irradiation greatly influenced the release behaviors of FLU and RHB from electrospun composite fibers, in which temperature played a more dominant role than the pure ultrasound effect. With ultrasound treatment, both the irradiation power and pulsed cycle had the effect on the drug release profiles, which could be utilized to achieve on-demand drug release. These results indicated that ultrasound irradiation was a versatile approach to realize the on-demand controlled release of two different drugs located in different part of the electrospun composite biomaterials, and this novel delivery system might be used as medical implant devices for a variety of biomedical applications, such as tumor treatment and insulin delivery.
